# OSM May Serve as a Biomarker of Poor Prognosis in Clear Cell Renal Cell Carcinoma and Promote Tumor Cell Invasion and Migration

**DOI:** 10.1155/2023/6665452

**Published:** 2023-11-21

**Authors:** Shuzhang Wei, Yin Chen, Xiaokai Shi, Li Zuo, Lifeng Zhang

**Affiliations:** ^1^Department of Urology, The Affiliated Changzhou No. 2 People's Hospital of Nanjing Medical University, Changzhou 213000, China; ^2^Department of Urology, Changzhou Second People's Hospital, Changzhou Medical Center, Nanjing Medical University, China; ^3^Department of Urology, Changzhou Seventh People's Hospital, China

## Abstract

**Background:**

Currently, the role of oncostatin M (OSM) in clear cell renal cell carcinoma (ccRCC) has not been investigated. This study will explore the impact of OSM on ccRCC expression, prognosis, and cell function.

**Materials and Methods:**

In this study, we used The Cancer Genome Atlas (TCGA) database to evaluate OSM expression characteristics, pathogenic factor distribution, and prognostic aspects in ccRCC. We also combined this analysis with qRT-PCR to verify OSM mRNA expression levels at the tissue level. Then, the effects of OSM on the proliferation, invasion, and migration abilities of ccRCC cells were explored through CCK8, Transwell, Western blotting, and immunofluorescence experiments. Finally, the oncogenic mechanisms associated with OSM in ccRCC were explored through signaling pathway enrichment and single-cell analysis.

**Results:**

The results demonstrated that OSM was significantly more expressed in ccRCC than in normal tissues. According to the survival analysis, OSM in ccRCC was considerably worse in the group with high expression than in the group with low expression. Also, the univariate and multivariate Cox analyses of clinical characteristics show that OSM in ccRCC may be able to predict a poor prognosis on its own as a biomarker. In vitro cellular experiments demonstrated that high OSM expression had no discernible impact on ccRCC cell proliferation compared to the control group, but it did promote tumor cell invasion and migration. Signaling pathways and single-cell analysis revealed that OSM might promote ccRCC invasion and migration through M2 macrophages.

**Conclusion:**

In conclusion, OSM may serve as an independent poor prognostic biomarker in ccRCC and promote tumor cell invasion and migration. This discovery is expected to provide a new therapeutic target for patients with recurrent and metastatic ccRCC.

## 1. Introduction

Renal cell carcinoma (RCC) is a prevalent malignant neoplasm affecting the urinary system, posing a global health burden. The International Agency for Research on Cancer has reported an alarming annual incidence of over 430,000 newly diagnosed cases of RCC worldwide [1]. Clear cell RCC (ccRCC) is the most common pathological subtype, accounting for approximately 70%-80% of all cases [2]. Due to the highly insidious and aggressive nature of ccRCC, approximately 30%-40% of patients receive their diagnosis at an advanced stage, resulting in a five-year survival rate of less than 12% among ccRCC patients [3–5]. Despite advancements in imaging technology, the identification of new therapeutic targets, and the clinical application of immunotherapy, ccRCC still accounts for 180,000 deaths annually worldwide [1, 6–9]. Consequently, finding reliable prognostic indicators and viable treatment targets for ccRCC remains ongoing.

Oncostatin M (OSM) is a secreted cytokine and growth regulator that belongs to the interleukin 6 cytokine family, one of the most important cytokine families in tumorigenesis and metastasis [10, 11]. This protein also regulates the production of other cytokines, including interleukin 6, granulocyte colony-stimulating factor, and granulocyte-macrophage colony-stimulating factor in endothelial cells [10]. Due to these biological properties of OSM, it is gradually being studied in terms of tumor proliferation, invasion, migration, and tumor microenvironment (TME). In breast cancer, OSM acts as a central regulator of crosstalk between immune cells, fibroblasts, and cancer cells in TME, promoting tumor cell progression and metastasis by regulating immune-stromal-cancer cell interactions [12]. In ovarian cancer, by stimulating STAT3 signaling, the OSM receptor contributes significantly to the proliferation and dissemination of tumor cells [13]. OSM stimulates inflammatory gene expression in pancreatic cancer-associated fibroblasts, shaping a TME suitable for tumor survival and contributing to the proliferation and metastasis of tumor cells [14]. Therefore, the study of OSM is essential for understanding the alterations of TME and tumor-targeted therapy.

In cultured renal fibroblasts, OSM has been demonstrated to induce upregulation of inflammatory cytokines. OSM potentially exerts a direct influence on renal tubular epithelial cells and renal fibroblasts, leading to the production of crystal-binding molecules and inflammatory cytokines. These factors may contribute to the formation of renal crystal deposits [15]. However, the specific role of OSM in ccRCC remains unexplored. In this study, we will explore the impact of OSM in ccRCC cells in terms of expression, prognosis, proliferation, invasion, and migration through bioinformatics combined with cellular experiments. Our study will contribute to identifying a novel target for the treatment of ccRCC, providing a basis for potential therapeutic interventions.

## 2. Materials and Methods

### 2.1. Public Data Acquisition and Analysis

In this study, we obtained the ccRCC mRNA expression dataset (tumor = 539, normal = 72) and clinicopathological parameters dataset from The Cancer Genome Atlas (TCGA; https://www.cancer.gov/) database, such as follow-up time, survival status, age, gender, total pathological stage, T stage, N stage, M stage, and pathological grading (Table 1). We performed unpaired and paired difference analyses using the “limma” R package to examine the variations in OSM expression between tumor and normal tissues. The default parameters of the “limma” package were utilized for our analysis (*P* < 0.05, |LogFC| > 1). We also carried out a survival analysis using the “survival” R package. Utilizing the “rms” program, OSM expression data and clinicopathological factors were combined to create the nomogram survival prediction scoring system.

### 2.2. qRT-PCR Analysis of OSM mRNA Expression in ccRCC

Tissue samples of ccRCC patients included in this study were obtained from individuals who underwent surgical procedures at Changzhou Second People's Hospital, Nanjing Medical University, from June 1, 2021, to October 1, 2021. As a result, 10 pairs of ccRCC tumor tissue samples and adjacent normal tissues were collected for this analysis (Table 1). TRIzol reagent (Invitrogen, USA) was used to extract the total RNA from the tissue samples. Following the usual procedures, the isolated RNA was then reverse-transcribed into cDNA. The RNA sequences used in this study are as follows:

The OSM RNA sequence is as follows:

Forward primer: 5′-TACTGCTCACACAGAGGACGCT-3′

Reverse primer: 5′-AGATCTGTCTGCTTCTGGAGCTG-3′

The GAPDH RNA sequence is as follows:

Forward primer: 5′-TGGCACCGTCAAGGCTGAGAA-3′

Reverse primer: 5′-TGGTGAAGACGCCAGTGGACTC-3′

### 2.3. ccRCC Cell Culture

The American Type Culture Collection (ATCC; Manassas, USA) provided the human ccRCC cell lines Caki-1 and 786-O in this study. The tumor cell lines were grown in high-sugar Dulbecco's Modified Eagle's Medium (DMEM; Gibco, USA), which was then boosted with 10% foetal bovine serum and 1% penicillin-streptomycin solution (ScienCell, USA). The cells were kept in a controlled setting with 5% CO_2_ at 37°C.

### 2.4. Cell Proliferation, Invasion, and Migration

The OSM and control groups were set up in Caki-1 and 786-O cell lines, respectively. Cells in the midlogarithmic growth phase and in good growth condition were harvested, and single-cell suspensions were made by applying cell culture medium, and each well was then seeded with 5 × 10^3^ cells. Cells underwent 24, 48, 72, and 96 h incubation.

A CCK8 experiment was carried out to evaluate the cancer cells' capacity for proliferation. Cell growth was monitored using a CCK8 kit (Beyotime, China) by adding 50 *μ*l of freshly prepared CCK8 solution to each well and continuing incubation for 4 hours. The optical density value was then determined using an enzyme-linked immunoassay reader at a wavelength of 450 nm. Three independent experiments were conducted for each experiment.

The Transwell assay was used to identify cancer cells' capacity for invasion and migration. The Matrigel matrix gel (Becton Dickinson, USA), frozen in the -20°C refrigerator, was left to liquefy overnight at 4°C. Liquefied Matrigel matrix gel was diluted as a working solution at a ratio of 1 : 5. The upper chamber surface of the Transwell membrane received this solution, and it was then incubated for 30 min at 37°C. In the Transwell, the lower chamber received 600 *μ*l of growth medium containing 10% FBS (Solarbio, USA), while the upper chamber received 100 *μ*l of cell suspension and 200 *μ*l of serum-free medium. The plate was subsequently incubated for 48 hours at a controlled temperature of 37°C with 5% CO_2_. After the incubation period, the samples were fixed with 95% alcohol for 5 min and then stained for 1 hour with a 4 g/l crystal violet solution. Subsequently, five random fields at 100x magnification were selected under a microscope. Finally, the cell counts were quantified by ImageJ software.

### 2.5. Western Blotting

Total protein was extracted from well-cultured tumor cells by adding RIPA cell protein lysate (Santa Cruz, USA). For protein quantification, the BCA protein quantification kit (Shengong, China) was used in a 96-well plate with three replicate wells for each sample. Using 10% SDS-PAGE and PVDF membranes from Millipore (USA), cell lysates were separated from cell proteins for 90 min. The membranes were subsequently blocked for an hour in PBS containing 5% skim milk. The above solutions were incubated with primary antibodies OSM (1 : 1000; Sino Biological, China), E-cadherin (1 : 2000; Cell Signaling Technology, USA), N-cadherin (1 : 1000; PL Laboratories, Canada), vimentin (1 : 1000; Sino Biological, China), and GAPDH (1 : 2000; Solarbio, China) at 4°C overnight. The membrane is washed the following day and incubated for two hours at room temperature with a horseradish peroxidase-coupled secondary antibody (1 : 3000; Southern Biotech, USA). Three independent experiments were conducted for each experiment. Finally, the band density was quantified by ImageJ software.

### 2.6. Immunofluorescence Analysis

The tumor cells were cultured in a 6-well plate and fixed using a 4% paraformaldehyde solution for 30 min. After fixation, the cells were treated with a membrane-breaking working solution obtained from Ribiology (China) for 10 min at room temperature. The cell well plates were then supplemented with primary antibodies directed against OSM, E-cadherin, and vimentin and incubated overnight at 4°C in a humid environment. The next day, an Alexa Fluor 555-labeled goat anti-mouse IgG antibody (from Invitrogen, USA) was applied for detection. The cells restained with DAPI staining solution and incubated at room temperature for 10 min; then, the phosphate-buffered saline (PBS) was removed. A Nikon (Japan) fluorescence microscope was used to take immunofluorescence pictures.

### 2.7. Gene Set Enrichment Analysis (GSEA)

GSEA software (version 4.2.1) was used to do an enrichment analysis to look into the role of OSM carcinogenesis-related pathways in ccRCC. Then, phenotype *h* (*h* = 83/178) was assigned to the upregulated gene set. In contrast, the downregulated gene collection was classified as belonging to phenotype *l* (*l* = 95/178), and the “h-versus-l” comparison was chosen to find enriched OSM-related pathways. Significant enrichment was considered for a normalized enrichment score > 1, normal *p* value < 0.05, and false discovery rate < 0.25.

### 2.8. Single-Cell Analysis

The Tumor Immune Single-cell Hub 2 (TISCH2; http://tisch.comp-genomics.org/) website collects human tumor scRNA-seq datasets from the Gene Expression Omnibus (GEO) and ArrayExpress. The TISCH2 platform was used for uniform processing and analysis of these datasets, facilitating comprehensive exploration of the tumor single-cell transcriptome and corresponding patient information. The robust platform TISCH2 facilitates the investigation of the tumor microenvironment (TME) in many cancer types by enabling thorough cell type annotations at the individual cell level [16].

### 2.9. Statistical Analysis

The data in this study were statistically analyzed using the GraphPad Prism program (version 9.0). The Wilcoxon rank sum test was used to look at variables with nonnormal distributions, while Student's *t*-test was used to assess the statistical significance between two groups for numerical data that followed a normal distribution. The Kruskal-Wallis test was utilized to compare the median ranks of three or more independent groups, whereas the log-rank test was used as a statistical hypothesis test to evaluate survival patterns between two groups. The *p* value of <0.05 was considered statistically significant.

## 3. Results

### 3.1. OSM Is Significantly Overexpressed in ccRCC

**Figure 1:**
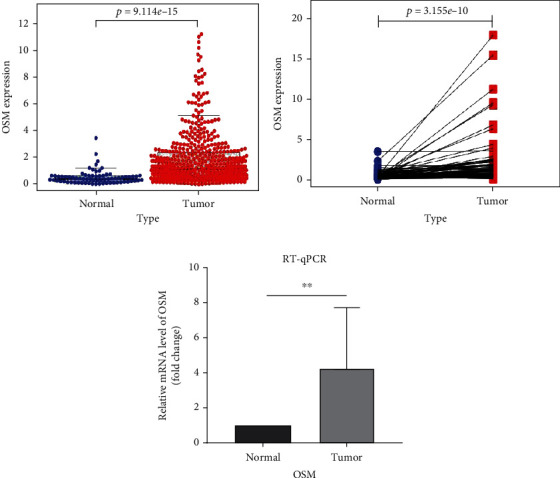
Analysis of OSM expression in ccRCC. (a, b) Paired and unpaired difference analysis of OSM in tumor and normal tissues in TCGA database. (c) Paired differential analysis of OSM expression in tumor tissues compared with paraneoplastic tissues in 10 cases by qRT-PCR (^∗∗^*p* value < 0.01).

Using the TCGA database, an unpaired differential analysis was used to examine the expression of OSM in ccRCC tumor tissues compared with normal tissues. The results showed that OSM expression was significantly higher in tumor tissues than in normal tissues (*p* value < 0.05; Figure 1(a)). Furthermore, paired difference analysis revealed a significant upregulation of OSM in ccRCC tumor tissues compared to their corresponding paraneoplastic tissues (*p* value < 0.05; Figure 1(b)). We used the qRT-PCR method to further analyze the RNA expression levels of OSM in 10 ccRCC tissues and associated paracancerous tissues. The results validated the above database findings that OSM was significantly more expressed in ccRCC tumor tissues when compared to matching paracancerous tissues (*p* value < 0.05; Figure 1(c)). In conclusion, OSM is significantly overexpressed in ccRCC.

### 3.2. Differential Distribution of OSM Expression in KRIC among Various Clinicopathological Parameters

Based on the median value of OSM expression in TCGA, the patient samples were divided into high and low expression groups. The results showed significant variations in the clinicopathological parameters T stage, M stage, total pathological stage, and pathological grading subtypes in the high and low OSM expression groups (*p* value < 0.05; Table 2).

**Figure 2:**
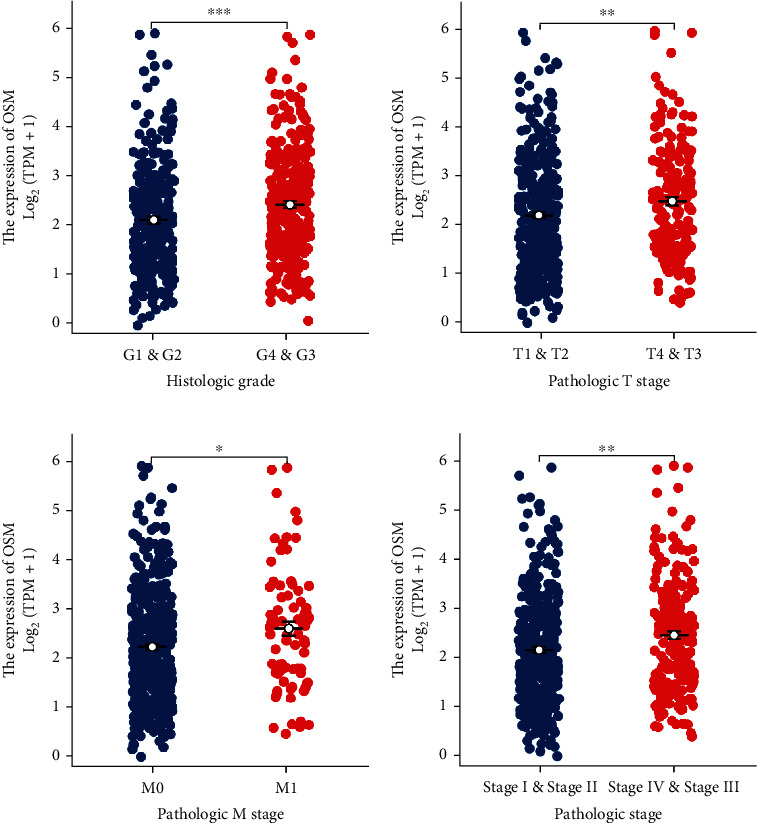
Differential expression analysis of OSM in clinicopathological parameters in ccRCC. Differential expression analysis of OSM in (a) pathological grading, (b) T stage, (c) M stage, and (d) total pathological stage. ^∗^*p* value < 0.05, ^∗∗^*p* value < 0.01, and ^∗∗∗^*p* value < 0.001.

We further analyzed the differential distribution of OSM in these clinicopathological parameters. According to the results, OSM expression was considerably higher in grades 3 and 4 than in grades 1 and 2 in histopathological grading (*p* value < 0.05; Figure 2(a)). Regarding histopathological stages, the expression of stage M1 was significantly higher than that of stage M0, the expression of stages T1 and T2 was significantly higher than that of stages T3 and T4, and the expression of stages III and IV was significantly higher than that of stages I and II (*p* value < 0.05; Figures 2(b)–2(d)). These findings showed that the elevated OSM expression in ccRCC might impact tumor development.

### 3.3. OSM May Serve as an Independent Prognostic Biomarker for Poor Prognosis in ccRCC

**Figure 3:**
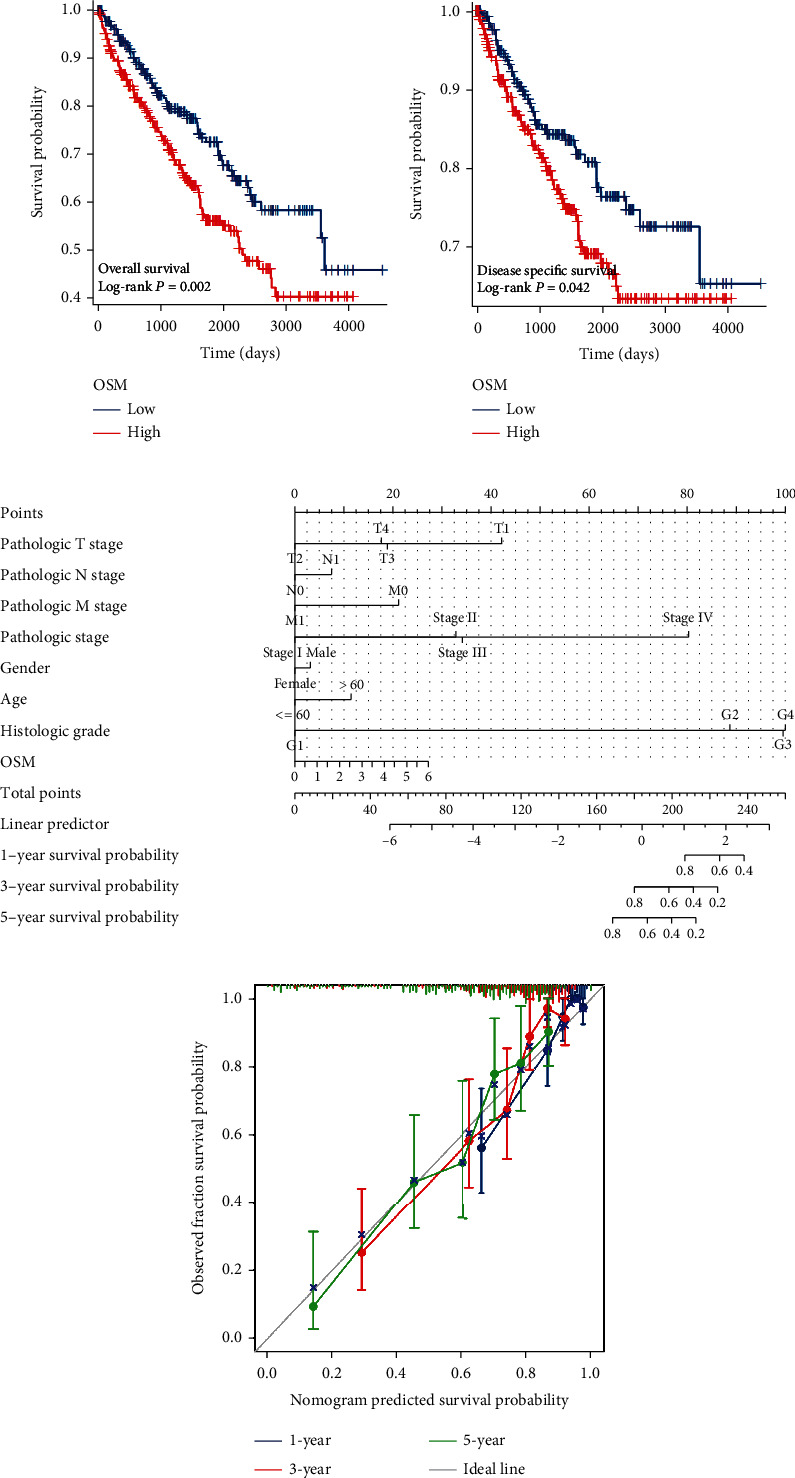
Prognostic analysis of OSM in ccRCC. Kaplan-Meier survival analysis method to compare the differences in (a) overall survival and (b) disease-specific survival prognostic indicators between the OSM high and low expression groups in the TCGA database (*p* value < 0.05). (c) Integration of OSM expression and clinicopathological parameters from the TCGA database to construct a nomogram survival prediction system to predict 1-, 3-, and 5-year survival rates in ccRCC patients. (d) Plotting nomogram prediction system calibration curves to assess patient prognostic predictive ability.

A survival study was carried out to investigate OSM's predictive function in ccRCC. The median OSM expression in the TCGA dataset was the basis for classifying the patients into high (*n* = 265) and low (*n* = 265) expression groups. The results demonstrated that the high OSM expression group had significantly worse overall survival and disease-specific survival than the low expression group (*p* value < 0.05; Figures 3(a) and 3(b)). Subsequently, the univariate and multivariate Cox prognostic analyses were performed by integrating OSM expression data in TCGA with clinicopathological parameters. OSM can act as an independent biomarker for a poor prognosis in ccRCC, according to the results of both univariate and multivariate Cox analyses (HR > 1, *p* value < 0.05; Table 3).

To predict the prognosis of patients with ccRCC, we assessed the clinical value of the characteristics based on univariate and multivariate Cox regression analyses, and eight clinical indicators, including gender, age, TNM stage, pathologic stage, pathological stage, and OSM expression, were selected to be included in the nomogram. Each factor in the prediction system corresponds to a score, and the sum of the scores for all clinical factors corresponds to the total patient score, thus predicting 1-, 3-, and 5-year survival rates (Figure 3(c)). A higher total number of points were associated with a worse prognosis. The survival prediction calibration curve revealed that the prediction curves fluctuated above and below the calibration curve, indicating that the survival prediction model we constructed had high accuracy (Figure 3(d)).

### 3.4. High OSM Expression Can Promote ccRCC Invasion and Migration

**Figure 4:**
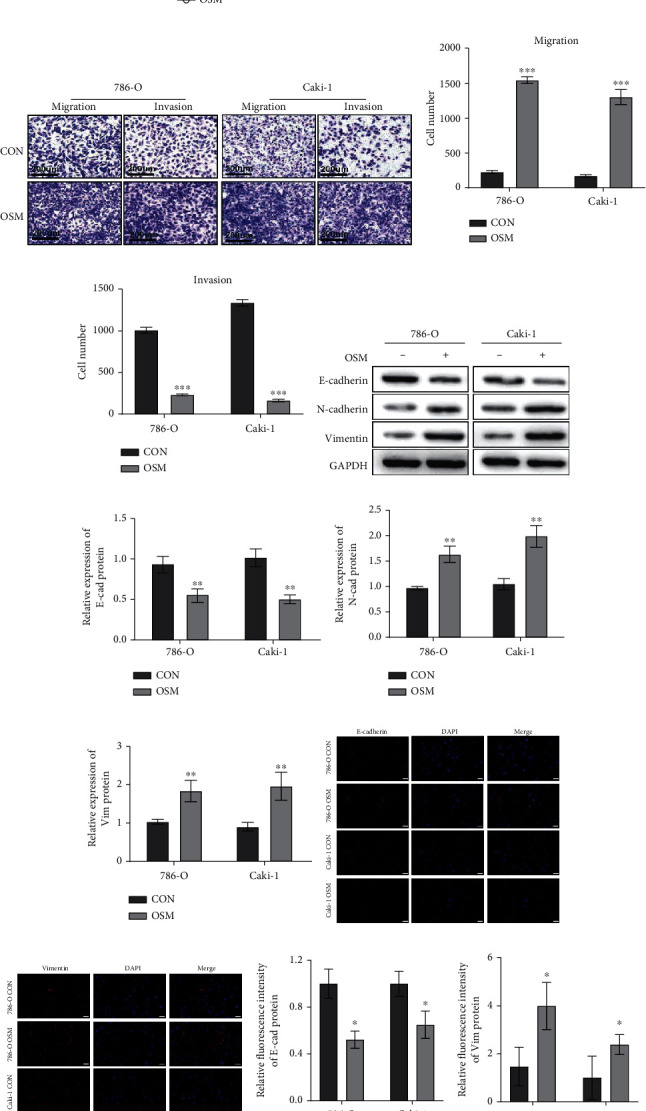
Analysis of the effect of OSM expression on the function of ccRCC cells. (a) Effect of the OSM high expression group and control group on the proliferation of ccRCC cells by using the CCK8 experiment (*p* value < 0.05). (b–d) Effect of the OSM high expression group versus the control group on ccRCC cell invasion and migration by using the Transwell experiment (*p* value < 0.05). (e–h) Effect of OSM expression in ccRCC on the expression of tumor metastasis-related molecules such as E-cadherin, N-cadherin, and vimentin by using Western blotting experiment. (i–l) Effect of OSM expression in ccRCC on the expression of E-cadherin and vimentin by immunofluorescence assay. Scale bar, 50 *μ*m. ^∗^*p* value < 0.05, ^∗∗^*p* value < 0.01, and ^∗∗∗^*p* value < 0.001.

The goal of the current study is to determine how OSM affects ccRCC tumor cells' functional behaviour at the cellular level. According to the results of the cell proliferation assay, there was no discernible difference in the proliferation of the ccRCC tumor cells between the control group and the group with high OSM expression, indicating that high OSM expression does not stimulate the proliferation of ccRCC tumor cells (Figure 4(a)). However, the cell invasion and migration assays demonstrated that the group with high OSM expression exhibited enhanced invasion and migration capabilities compared to the control group, and quantitative analysis confirmed the statistical significance of this difference (*p* value < 0.05; Figures 4(b)–4(d)). These findings suggest a potential association between OSM and ccRCC tumor recurrence and metastasis.

To further validate the above conclusions, we selected molecules associated with tumor cell adhesion, invasion, and metastasis and examined their relationship with OSM. Studies have shown that tumor invasion and migration are significantly influenced by altered E-cadherin expression and elevated N-cadherin and vimentin expression [17–20]. When compared to the control group, Western blotting assays showed a decrease in the expression of E-cadherin and an increase in the expression of N-cadherin and vimentin, and quantitative analysis indicated that this differential expression was significant (*p* value < 0.05; Figures 4(e)–4(h)). Immunofluorescence staining investigations further confirmed these reliable results, showing a decrease in E-cadherin distribution and a rise in vimentin distribution in the group with high OSM expression compared to the control group (Figures 4(i)–4(l)). In conclusion, high OSM expression in ccRCC can promote tumor cell proliferation and migration.

### 3.5. OSM May Promote Tumor Metastasis by Regulating Macrophages

**Figure 5:**
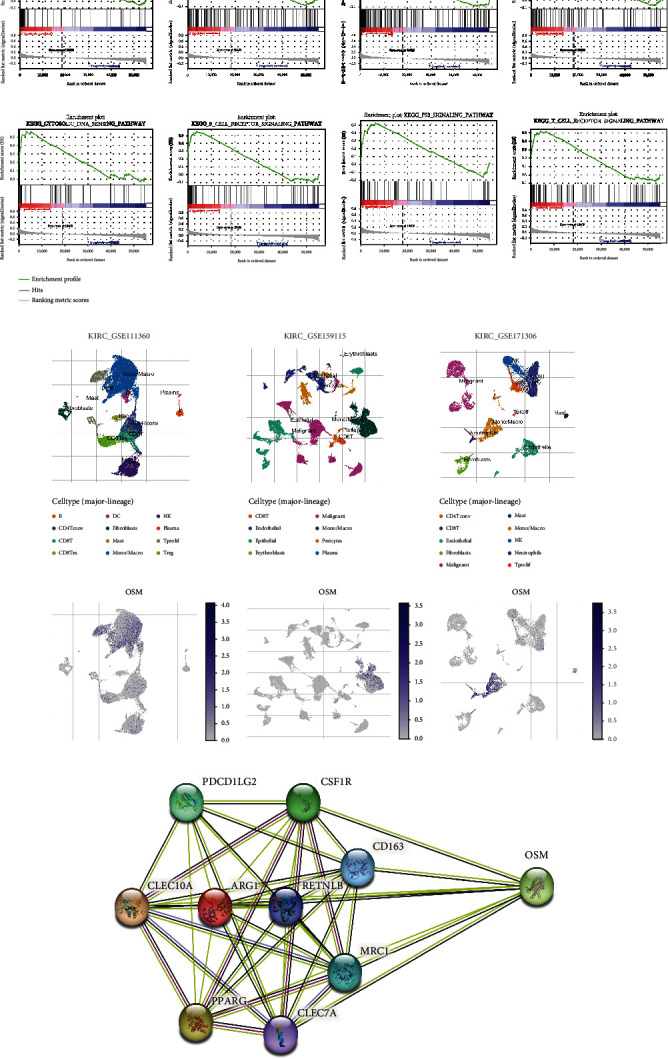
Analysis of the mechanism of OSM carcinogenesis in ccRCC. (a) GSEA of the signaling pathway upregulated by OSM in ccRCC. (b) Single-cell analysis of OSM expression-associated immune cell subpopulations in ccRCC. (c) Protein interaction analysis of OSM with M2 macrophage surface markers.

The top 8 signaling pathways upregulated by OSM in ccRCC were screened by GSEA (Table 4). The results indicated that OSM upregulated immune signaling pathways such as NOD-like receptor, chemokine signaling, Toll-like receptor signaling, B-cell receptor signaling, and T cell receptor signaling (Figure 5(a)). Previous studies have demonstrated the importance of these immune signaling pathways in tumor killing [21–24]. However, high OSM expression in ccRCC upregulates these signaling pathways leading to a poor prognosis (Figure 5(a)). Therefore, high expression of OSM in ccRCC may mediate a state of TME immunosuppression and thus promote tumor progression.

Then, we selected three datasets from different primary renal clear cell tumors (all untreated). These datasets are referred to as the GSE111360, GSE159115, and GSE171306 datasets of ccRCC, and we performed single-cell analyses on them using the TISCH website. The results revealed that OSM expression was mainly located in the monocyte-macrophage subpopulation of ccRCC (Figure 5(b)). Macrophages have M1 and M2 states in response to stimulation, with M1 macrophages having a proinflammatory effect and M2 macrophages having an immunosuppressive effect [25]. Tumor cells can activate M2 macrophages, which encourage tumor angiogenesis, ease the invasion and migration of tumor cells, and reduce tumor immunity [26–28]. Currently, several studies have demonstrated that PDCD1LG2, CSF1R, MRC1, PPARG, ARG1, CD163, CLEC10A, CLEC7A, and RETNLB can be used as biomarkers on the surface of M2 macrophages [29–31]. Analysis using the STRING website (https://cn.string-db.org/) revealed that OSM has protein interactions with M2 macrophage surface markers. Therefore, we suggest that OSM forms immunosuppression through M2 macrophages, thereby promoting ccRCC invasion and migration.

## 4. Discussion

Currently, ccRCC is a severe threat to human health, and approximately hundreds of thousands of ccRCC patients die each year worldwide [1]. With advances in medical technology, early surgical treatment remains the preferred option for ccRCC patients, while researchers have made progress in studying the molecular and cellular aspects of tumors, which has opened up new avenues for treatment.

The molecular therapy for ccRCC has transitioned from gene-targeted therapy to immunotherapy [32]. Targeted agents, such as sorafenib, pazopanib, and sunitinib, which inhibit vascular endothelial growth factor and its receptors, have been utilized in the treatment of ccRCC, and inhibition of rapamycin complex 1 by everolimus and temsirolimus has shown some success in improving the prognosis of patients with advanced stage [33, 34]. Nevertheless, there are still some ccRCC patients who do not benefit from it. The clinical use of immunotherapy offers new hope for patients with ccRCC. In 2015, the monoclonal antibody nivolumab, which targets the programmed death 1 (PD-1) immune checkpoint, received approval as a monotherapy for treating kidney cancer [32]. Subsequently, the clinical use of an increasing number of immunotherapeutic agents and their combinations has greatly improved the long-term prognosis of ccRCC patients [34, 35]. RCC is an immunogenic cancer that immune cells frequently infiltrate. During tumorigenesis and growth stages, renal cell carcinoma cells induce the expression of cytokines in the TME, leading to a state of tumor immunosuppression and promoting immune escape. Tumor-associated immunosuppressive cells play a role in tumor growth, metastasis, and invasion [36]. Single-cell analyses show that OSM is mainly expressed in monocyte macrophages, which are the most abundant immune-associated infiltrating stromal cells in and around tumors and exhibit different phenotypes and functions. The immune escape mechanism of renal cell carcinoma includes various aspects, but the specific molecular mechanism is still difficult to elucidate. Therefore, clarifying the immune escape mechanism of renal cell carcinoma can lay a solid foundation for identifying suitable molecular targets, which can significantly improve the clinical efficacy of RCC treatment.

Using the TCGA dataset and clinical samples, we found that OSM was overexpressed in ccRCC and could be an independent biomarker of a bad prognosis. Cell function experiments demonstrated that high OSM expression in ccRCC could promote tumor cell invasion and migration. Therefore, targeting OSM may hold promise as a potential therapeutic strategy for addressing ccRCC metastasis and recurrence. Studies have demonstrated that OSM overexpression in hepatocellular carcinoma can promote tumor cell invasion and angiogenesis [37]. In breast cancer, OSM is highly expressed in ductal carcinoma in situ and can promote lung metastasis [38]. In gastric cancer, OSM promotes gastric cancer growth and metastasis through STAT3/FAK/Src signaling [39]. OSM has also been demonstrated to function as a cancer prognostic marker. In cholangiocarcinomas, OSM expression controls immune cell survival and tumor invasion. It can be utilized as a potential biomarker and therapeutic target for cholangiocarcinoma prognosis [40]. By regulating the hepatic inflammatory milieu, OSM also contributes significantly to the development of hepatocarcinogenesis. The expression of OSM in hepatocellular carcinoma peritumoral tissues is positively connected with overall patient survival [41]. In summary, OSM has metastasis-promoting properties in a variety of tumors. Therefore, research targeting OSM has a significant perspective.

We also revealed by pathway enrichment and single-cell analysis that OSM may promote tumor metastasis through M2 macrophages, which are essential for tumor growth, metastasis, immune regulation, tumor angiogenesis, TME remodelling, and response to cancer therapy [25, 42, 43]. The study demonstrates that the CHI3L1 protein secreted by tumor-recruiting M2 macrophages promotes metastasis in gastric and breast cancers [44]. Macrophages activated by histamine receptor H1 can differentiate towards M2-like immunosuppressive phenotypes with increased expression of the immune checkpoint VISTA, resulting in dysfunctional T cells and thus TME immunosuppression [45]. In lung cancer, tumor-associated M2 macrophages interact with USP7 to regulate the antitumor immune response [46]. Therefore, studies targeting OSM in ccRCC may provide new directions for immunotherapy in ccRCC patients.

However, there are some things that could be improved in our study. For example, our experimental studies were only at the in vitro cellular level and were not further validated in vivo. Because OSM promotes ccRCC cell invasion and migration, we have only analyzed the possibility in terms of single-cell data and lack further specific regulatory mechanisms. This will be investigated in more depth in our subsequent research projects.

In conclusion, OSM can facilitate tumor cell invasion and migration and may act as a standalone biomarker for predicting poor prognosis in ccRCC. For patients with recurrent and metastatic ccRCC, this discovery may offer a novel treatment target.

## Figures and Tables

**Table 1:** Information on the patient sample included in this study.

Characteristics	TCGA	Clinical samples
*n*	537	10
Pathologic T stage, *n* (%)	537	10
T1-2	344 (64.1%)	6 (60.0%)
T3-4	193 (35.9%)	1 (10.0%)
Pathologic N stage, *n* (%)	257	5
N0	240 (93.4%)	5 (100.0%)
N1	17 (6.6%)	0 (0.0%)
Pathologic M stage, *n* (%)	505	9
M0	426 (84.4%)	8 (88.9%)
M1	79 (15.6%)	1 (11.1%)
Pathologic stage, *n* (%)	534	10
Stage I-II	326 (61.0%)	8 (80.0%)
Stage III-IV	208 (39.0%)	2 (20.0%)
Gender, *n* (%)	537	10
Female	191 (35.6%)	4 (40.0%)
Male	346 (64.4%)	6 (60.0%)
Age, *n* (%)	537	10
≤60	266 (49.5%)	4 (40.0%)
>60	271 (50.5%)	6 (60.0%)
Histologic grade, *n* (%)	529	9
G1-2	244 (46.1%)	5 (55.6%)
G3-4	285 (53.9%)	4 (44.4%)

**Table 2:** Differential distribution of clinicopathological parameters in high and low OSM expression groups.

Characteristics	Low expression of OSM	High expression of OSM	*p* value
*n*	265	265	
Pathologic T stage, *n* (%)			0.019
T1-2	183 (34.5%)	157 (29.6%)	
T3-4	82 (15.5%)	108 (20.4%)	
Pathologic N stage, *n* (%)			0.151
N0	119 (46.7%)	120 (47.0%)	
N1	5 (2.0%)	11 (4.3%)	
Pathologic M stage, *n* (%)			0.017
M0	218 (43.8%)	202 (40.6%)	
M1	29 (5.8%)	49 (9.8%)	
Pathologic stage, *n* (%)			0.011
Stage I-II	175 (33.2%)	147 (27.9%)	
Stage III-IV	88 (16.7%)	117 (22.2%)	
Gender, *n* (%)			0.203
Female	100 (18.9%)	86 (16.2%)	
Male	165 (31.1%)	179 (33.8%)	
Age, *n* (%)			0.082
≤60	122 (23.0%)	142 (26.8%)	
>60	143 (27.0%)	123 (23.2%)	
Histologic grade, *n* (%)			0.002
G1-2	137 (26.2%)	104 (19.9%)	
G3-4	121 (23.2%)	160 (30.7%)	

**Table 3:** Univariate and multivariate Cox analyses of OSM in ccRCC.

Characteristics	Total (*N*)	HR (95% CI) univariate analysis	*p* value univariate analysis	HR (95% CI) multivariate analysis	*p* value multivariate analysis
Pathologic T stage	530				
T1+2	340	Reference		Reference	
T3+4	190	3.321 (2.356-4.681)	<0.001	0.412 (0.103-1.642)	0.209
Pathologic N stage	255				
N0	239	Reference		Reference	
N1	16	3.422 (1.817-6.446)	<0.001	1.364 (0.447-4.160)	0.586
Pathologic M stage	498				
M0	420	Reference		Reference	
M1	78	4.401 (3.226-6.002)	<0.001	0.433 (0.040-4.726)	0.493
Pathologic stage	527				
Stage I-II	322	Reference		Reference	
Stage III-IV	205	2.649 (1.767-3.971)	<0.001	3.962 (0.927-16.926)	0.063
Histologic grade	522				
G1-2	241	Reference		Reference	
G3-4	281	2.253 (1.835-2.766)	<0.001	1.528 (1.210-1.929)	<0.001
Age	530				
≤60	264	Reference		Reference	
>60	266	1.791 (1.319-2.432)	<0.001	1.595 (1.030-2.470)	0.037
Gender	530				
Female	187	Reference			
Male	354	0.924 (0.679-1.257)	0.613		
OSM	530				
Low	265	Reference		Reference	
High	265	1.607 (1.185-2.178)	0.002	1.717 (1.094-2.695)	0.019

**Table 4:** Signaling pathways upregulated by OSM in ccRCC.

Pathway	Normalized enrichment score	Nominal *p* value	False discovery rate *q* value
NOD_LIKE_RECEPTOR_SIGNALING	1.7800	0.0000	0.0003
JAK_STAT_SIGNALING	2.1702	0.0000	0.0053
CHEMOKINE_SIGNALING	2.1596	0.0020	0.0050
TOLL_LIKE_RECEPTOR_SIGNALING	2.1522	0.0000	0.0050
CYTOSOLIC_DNA_SENSING	2.0095	0.0020	0.0161
B_CELL_RECEPTOR_SIGNALING	1.9733	0.0079	0.0200
P53_SIGNALING	1.8893	0.0178	0.0309
T_CELL_RECEPTOR_SIGNALING	1.8739	0.0158	0.0333

## Data Availability

The data for this study are available from the following databases: The Cancer Genome Atlas (https://www.cancer.gov/), TISCH2 (http://tisch.comp-genomics.org/), and STRING (https://cn.string-db.org/).

## Abbreviations

OSM: Oncostatin M
TCGA: The Cancer Genome Atlas
RCC: Renal cell carcinoma
ccRCC: Clear cell RCC
TME: Tumor microenvironment
GSEA: Gene set enrichment analysis
TISCH2: Tumor Immune Single-cell Hub 2.
